# Proteomic Identification of *Bombyx mori* Organelles Using the Engineered Ascorbate Peroxidase APEX and Development of Silkworm Organelle Proteome Database (SilkOrganPDB)

**DOI:** 10.3390/ijms22095051

**Published:** 2021-05-10

**Authors:** Tian Li, Chen Xu, Jinzhi Xu, Jian Luo, Bin Yu, Xianzhi Meng, Chunfeng Li, Guoqing Pan, Zeyang Zhou

**Affiliations:** 1 State Key Laboratory of Silkworm Genome Biology, Southwest University, Chongqing 400715, China; xuchen_swu@163.com (C.X.); xjz_swu@126.com (J.X.); jianluo0214@163.com (J.L.); yubin5868@outlook.com (B.Y.); mxzmddswu@126.com (X.M.); cflil@163.com (C.L.); gqpan@swu.edu.cn (G.P.); 2 Chongqing Key Laboratory of Microsporidia Infection and Control, Southwest University, Chongqing 400715, China; 3 College of Life Science, Chongqing Normal University, Chongqing 400047, China

**Keywords:** silkworm, *Bombyx mori*, organelle, proteome, database

## Abstract

Silkworm *Bombyx mori* is an economically important insect and a lepidopteran model. Organelle proteome is vital to understanding gene functions; however, it remains to be identified in silkworm. Here, using the engineered ascorbate peroxidase APEX, we constructed transgenic *B. mori* embryo cells (BmE) expressing APEX-NLS, COX4-APEX, APEX-Rev, and APEX-KDEL in nucleus, mitochondrial matrix (MM), cytosol, and endoplasmic reticulum (ER), and isolated the biotin-labeled proteins using streptavidin-affinity purification, respectively. The isolated proteins were determined using LC-MS/MS and annotated by searching *B. mori* genomes downloaded from GenBank, SilkBase, SilkDB 2.0, and SilkDB 3.0, resulting in 842, 495, 311, and 445 organelle proteins identified, respectively. We mapped the 296 MM proteins annotated in the GenBank data to mitochondrial protein databases of the fly, human, and mouse, and found that 140 (47%) proteins are homologous to 80 fly proteins, and 65 (22%) proteins match to 31 and 29 human and mouse proteins, respectively. Protein orthology was predicted in multiple insects using OrthoMCL, producing 460 families containing 839 proteins we identified. Out of 460 families, 363 were highly conserved and found in all insects, leaving only three proteins without orthology in other insects, indicating that the identified proteins are highly conserved and probably play important roles in insects. A gene ontology enrichment analysis by clusterProfiler revealed that the nucleus proteins significantly enriched in cellular component terms of nucleus and nucleolus, the MM proteins markedly enriched in molecular function terms of nucleotide binding, and the cytosol proteins mainly enriched in biological process terms of small molecule metabolism. To facilitate the usage and analysis of our data, we developed an open-access database, Silkworm Organelle Proteome Database (SilkOrganPDB), which provides multiple modules for searching, browsing, downloading, and analyzing these proteins, including BLAST, HMMER, Organelle Proteins, Protein Locations, Sequences, Gene Ontology, Homologs, and Phylogeny. In summary, our work revealed the protein composition of silkworm BmE organelles and provided a database resource helpful for understanding the functions and evolution of these proteins.

## 1. Introduction

Silk moth is a large group of lepidopteran insects that includes the domestic silkworm *Bombyx mori* and some wild animals such as *Actias selene*, *Antheraea assamensis*, *Antheraea pernyi*, *Bombyx huttoni*, *Bombyx mandarina*, *Caligula japonica*, *Eriogyna pyretoum*, *Philosamia cynthiaricini*, and *Samia cynthia*. The *B. mori* is an economically important insect and has become a model organism for lepidoptera. To date, three silkworm genomes from the *B. huttoni*, *B. mandarina*, and *B. mori* have been sequenced and deposited in the GenBank database under the accession numbers GCA_002197625.1, GCA_003987935.1, and GCA_000151625.1, respectively. The genomic and genetic maps greatly promoted studies of the functional and comparative genomics of silkworms [1,2,3], especially with a high-quality genome assembly of *B. mori* obtained and published recently [4]. This improved assembly and annotation provided a more accurate reference for transcriptomics and proteomics studies. Referring to the genome sequence, large-scale transcriptome analysis of silkworms was performed with microarray and RNA-seq technologies, respectively [5,6]. Transcriptional profiles in different tissues and at different development stages partially indicate the functional characteristics of silkworm protein-coding genes; however, these need to be further revealed at the proteomics level. Silkworm proteomes have been further dissected at the organ and tissue levels. Proteins from the head, silk gland, colleterial gland, hemolymph, midgut, embryo, and spinneret have been isolated and identified with mass spectrometry [7,8,9,10,11,12,13,14,15,16]. In addition, silkworm proteomic responses against infections by virus, bacteria, and fungi pathogens have also been widely investigated using high-throughput methods [17,18,19,20,21,22]. To date, little is known about the protein composition of silkworm subcellular organelles like the nucleus, mitochondria, cytosol, and endoplasmic reticulum (ER), except that Wang et al. isolated proteins from the cytosol, mitochondria and microsomes of silkworm infected by nucleopolyhedrovirus [23]. However, organelle proteomes that identified with targeted and accurate isolation methods have not been reported in silkworms.

Studies of organelle proteome have been limited by the technology for specific purifying of objective organelles from cells and tissues. Recently, a proteomic mapping technology via spatially restricted enzymatic tagging using the engineered ascorbate peroxidase APEX was developed and successfully used to identify organelle proteins from living cells and tissues [24,25,26,27]. Proteins from human mitochondrial matrix (MM), intermembrane space, membranes of ER and outer mitochondria, and lipid droplets, fly tissues, yeast, and worm organelles have been identified using the engineered APEX [28,29,30,31,32]. The APEX-based technology has become an effective tool for identifying proteomes in cells and tissues.

Here, for the first time, we isolated and identified protein components from the nucleus, MM, ER, and cytosol of silkworm embryo cells (BmE), and developed an open-access database resource for silkworm organelle proteomics.

## 2. Results

### 2.1. Expression of APEX in Transgenic BmE cells

**Figure 1:**
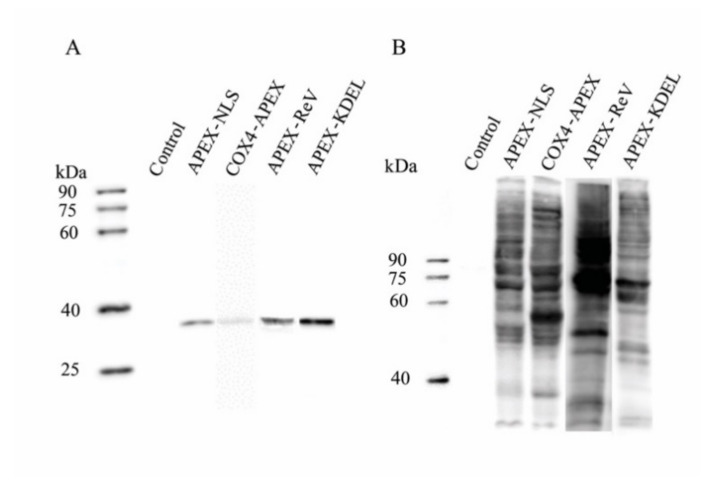
Western blotting analysis of APEX expression and APEX-mediated protein biotinylation. (**A**) Western blotting analysis of the expression of APEX with anti-FLAG. (**B**) Western blotting analysis of APEX-mediated biotinylation in transfected BmE cells using streptavidin. The control was the BmE cells without APEX-transfection.

Transgenic BmE cells expressing APEX fused with a leading peptide including COX4, NLS, Rev, and KDEL were made for translocating the APEX in MM, nucleus, cytosol, and ER, respectively. For verifying the expression and subcellular localization of the APEX, a FLAG tag was inserted between the APEX and leading peptide. The expression of COX4-FLAG-APEX, APEX-FLAG-NLS, APEX-FLAG-Rev, and APEX-FLAG-KDEL was determined using Western blotting with an antibody against the FLAG (anti-FLAG). As a result, the fusion proteins were expressed explicitly in transgenic cells (Figure 1A).

**Figure 2:**
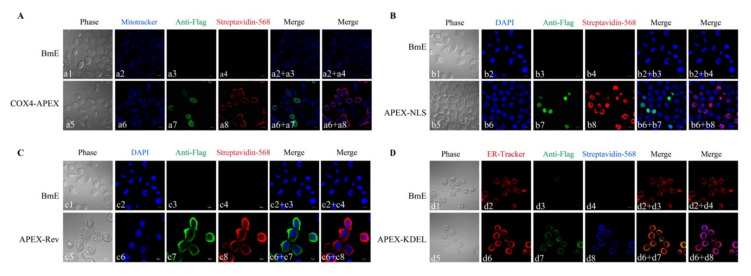
IFA of the subcellular localization of COX4-APEX (**A**), APEX-NLS (**B**), APEX-Rev (**C**), APEX-KDEL (**D**), and biotinylated proteins in BmE organelles, respectively. The BmE cells in a1-a4, b1-b4, c1-c4, d1-d4 were transfected without APEX (control). The BmE cells in a5-a8, b5-b8, c5-c8 and d5-d8 were transfected with COX4-APEX, APEX-NLS, APEX-Rev, and APEX-KDEL, respectively. The bar in A, B, and D indicates 10 μm, and the bar in C represents 5 μm.

The subcellular localization of APEX in transgenic BmE cells was analyzed using an indirect immunofluorescence assay (IFA) with anti-FLAG. The COX4-APEX was colocalized with mitochondria stained by MitoTracker Deep Red (Figure 2A), indicating that the COX4 specifically led the APEX into the mitochondria. The APEX-NLS colocalized with the nuclear dye DAPI (sigma) (Figure 2B), APEX-Rev localized in the cytosol (Figure 2C), and the APEX-KDEL colocalized with the ER dye (Thermo Fisher) (Figure 2D). These results manifested that the APEX was successfully expressed in BmE cells, and the leading sequences could specifically translocate the APEX into target organelles.

### 2.2. Verification of APEX-Mediated Protein Biotinylation

The catalytic activity of the APEX in the BmE organelles was verified by adding reaction substrates, biotin-phenol, and H_2_O_2_ into the culture media. The biotinylation was demonstrated using Western blotting with an antibody against streptavidin-HPR (Figure 1B). In the result, protein biotinylation was found in APEX-transgenic BmE cells, but not in that without APEX transfection. To examine the subcellular localization of the biotinylated proteins, Alexa Flour 568 conjugated streptavidin was used to perform IFA, which results showed that the biotinylated proteins well overlapped with COX4-APEX in MM (Figure 2A), co-localized with APEX-NLS in the nucleus (Figure 2B), and merged with APEX-Rev and APEX-KEDL in cytosol and ER, respectively (Figure 2C, D). These results indicated that the APEX successfully transferred biotin-phenol into biotin-radicals, leading to the specific biotinylation of proteins in targeted organelles.

### 2.3. Enrichment of the Biotinylated Proteins

To improve the accuracy of protein identification, we prepared two negative control groups and one experimental group, with three independent replicates for each group. One negative control group was BmE cell only expressing EGFP with the addition of substrates biotin-phenol and H_2_O_2_, and the other was BmE cell expressing APEX without the substrates. Thus, there should be no biotinylated proteins found in the two negative control groups. The experimental group was BmE cell expressing APEX and with the addition of reaction substrates, which should produce biotinylated proteins around where there was APEX protein.

**Figure 3:**
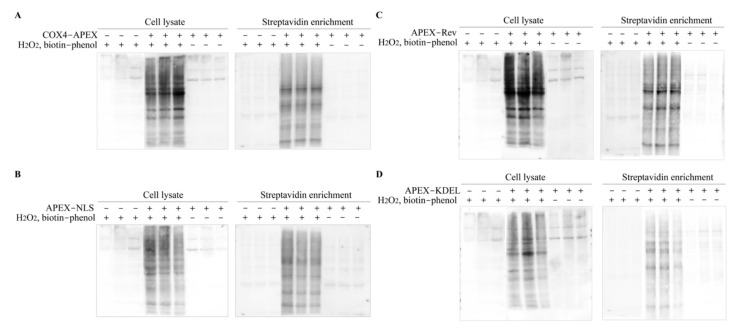
Streptavidin blotting analysis of biotinylated proteins before (cell lysate) and after enrichment (streptavidin enrichment) from MM (**A**), nucleus (**B**), cytosol (**C**) and ER (**D**).

Before the enrichment of the biotinylated proteins, we first determined the biotinylation in whole-cell lysate using Western blotting with streptavidin-HRP (Figure 3A–D). As a result, no biotin-labeled protein was detected in the negative control groups, while abundant biotinylated proteins were found in the experimental groups. Moreover, a significant difference in biotinylation was observed among the samples from the nucleus, MM, cytosol, and ER, suggesting that proteins in targeted organelles were successfully labeled by biotin. The biotinylated proteins were enriched using streptavidin-coated magnetic beads and then verified by Western blotting, the results of which manifested the successful enrichment (Figure 3A–D).

### 2.4. Identification and Annotation of the Biotinylated Proteins

**Figure 4:**
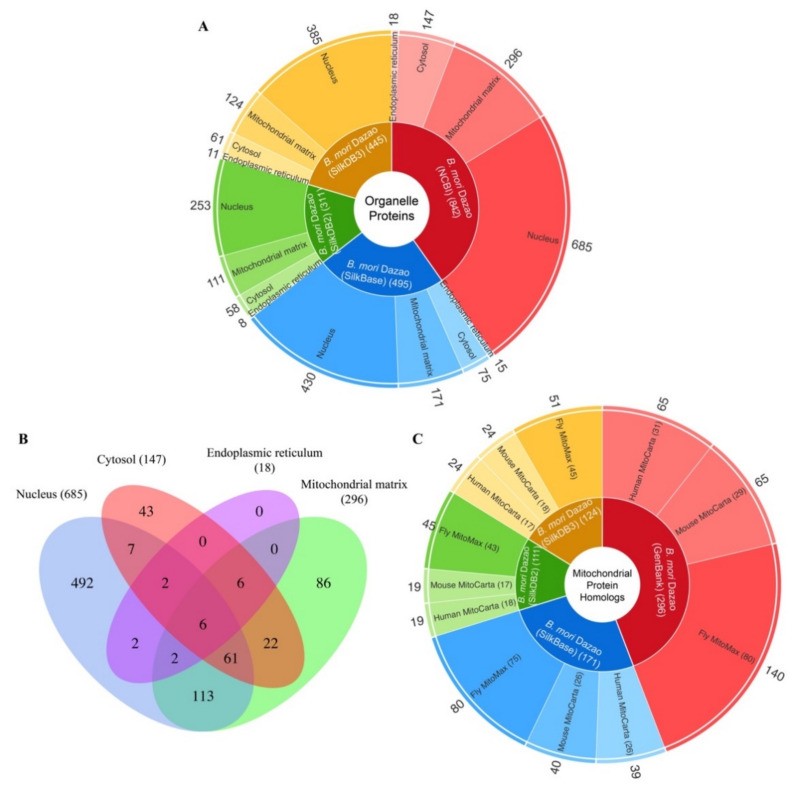
Summary of the identified silkworm organelle proteins. (**A**) The nucleus, MM, cytosol, and ER proteins are annotated in silkworm genomes from GenBank (red), SilkBase (blue), SilkDB 2.0 (green), and SilkDB 3.0 (yellow), respectively. (**B**) The distribution of the 842 organelle proteins annotated in GenBank data in the nucleus, cytosol, MM, and ER. (**C**) The identified silkworm MM proteins were mapped to databases of the fly MitoMax, mouse and human MitoCarta, in which proteins were mainly identified using the APEX method.

The enriched proteins from the nucleus, MM, cytosol, and ER were determined using mass spectrometry and searched against silkworm genomes, respectively. In general, we identified 842, 495, 311, and 445 proteins in genomes from GenBank, SilkBase, SilkDB 2.0, and SilkDB 3.0, respectively (Figure 4A, Table S1–S4). The largest number of proteins was obtained from the GenBank data, resulting in 685, 296, 147, and 18 proteins of the nucleus, MM, cytosol, and ER, respectively. Some proteins identified in the four organelles showed multiple subcellular localizations. For example, 193 out of 685 (28.2%) nucleus proteins and 210 out of 296 (70.9%) MM proteins were found in multiple organelles (Figure 4B).

The identified MM proteins were aligned against the fly MitoMax, human and mouse MitoCarta databases, in which mitochondrial proteins were mainly identified by the APEX strategy. For the MM proteins annotated in the genome of the GenBank version, 140 out of 296 (47%) proteins were aligned to 80 fly mitochondrial proteins, and 65 out of 296 (22%) proteins were matched to 31 and 29 human and mouse mitochondrial proteins, respectively (Figure 4C), indicating that the identified MM proteins are more conserved between silkworm and fly.

**Figure 5:**
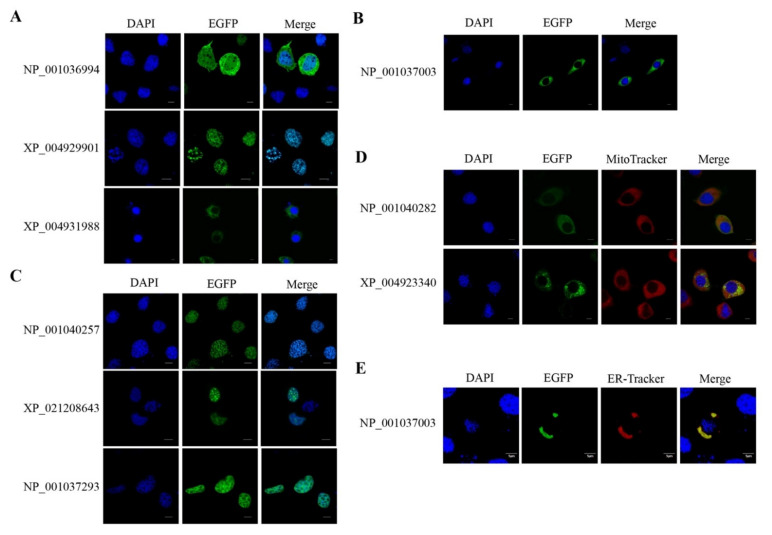
Verification of protein locations in BmE cells. The identified proteins were fused with EGFP and transfected BmE cells. The nucleus, MM, and ER were stained with DAPI (blue), MitoTracker (red) and ER-Tracker (red), respectively. (**A**) Subcellular localization of proteins identified in nucleus and cytosol. (**B**–**D**) Subcellular localization of proteins identified in the cytosol, nucleus, and MM, respectively. (**E**) Subcellular localization of protein identified in ER. The IDs indicate the GenBank accession number for each protein.

To verify the subcellular localization of the identified proteins, we performed transgenic analysis on some candidates in the BmE cells, including one protein from the cytosol and nucleus, two proteins from the cytosol, four proteins in the nucleus, and two proteins located in the mitochondrion, respectively. As shown in Figure 5, protein candidates fused with EGFP were shown in organelles where they were isolated from. These results indicated the high accuracy of our isolation and identification.

### 2.5. Orthology of the Organelle Proteins

**Figure 6:**
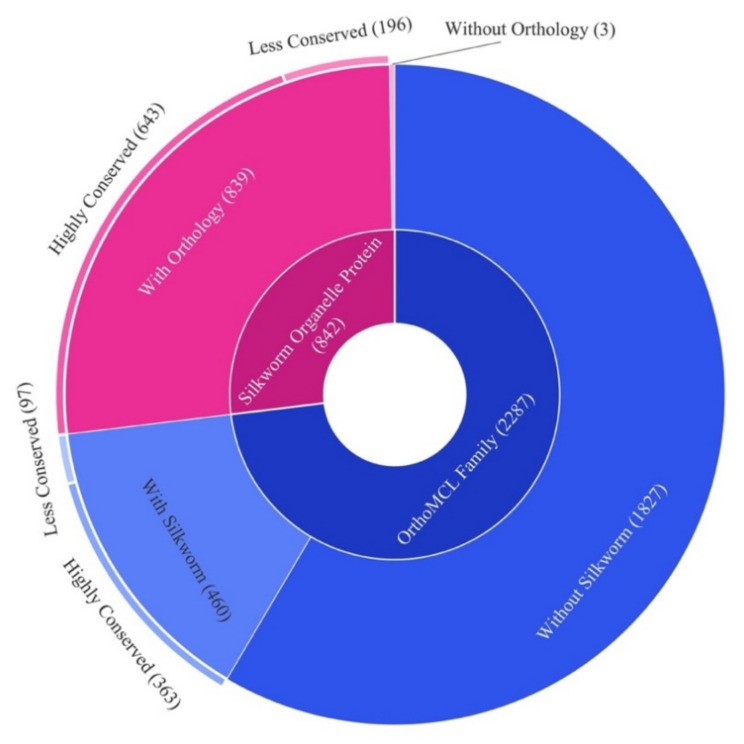
Summary of orthology for the identified silkworm organelle proteins annotated in the GenBank data. Pies in blue show the summary of protein families. Pies in magenta indicate the summary of silkworm organelle proteins included in families.

The protein orthology among B. mori, Bombyx mandarina, Manduca sexta, Papilio xuthus, Spodoptera litura, Trichoplusia ni, Tribolium castaneum, Aedes aegypti, Drosophila melanogaster, Apis cerana, and Apis mellifera was determined using the OrthoMCL. In the result, all proteins were grouped into 22,877 families, in which 460 families contained 839 out of 842 (99.6%) organelle proteins annotated in the GenBank silkworm data, leaving only 3 proteins without homolog in other insects. Moreover, 363 out of 460 (78.9%) families were found in all insects (Figure 6, Tables S5 and S6). These results suggest that the identified organelles proteins are highly conserved and probably play important roles in insects.

### 2.6. Gene Ontology Enrichment of the Identified Proteins

**Figure 7:**
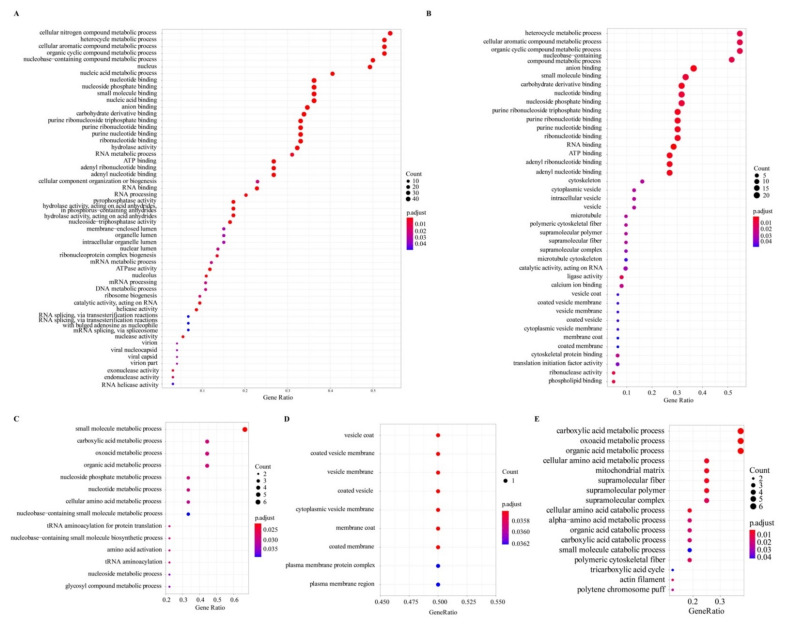
GO enrichment for silkworm organelle proteins of the nucleus (**A**), MM (**B**), cytosol (**C**), ER (**D**), and MitoMax homologs (**E**).

The organelle proteins annotated from the silkworm genome of the GenBank version were used to perform gene ontology (GO) enrichment analysis using clusterProfiler software. In the result, we obtained 53, 41, 20, and 9 GOs for 117, 54, 7, and 2 proteins of the nucleus, MM, cytosol, and ER, respectively. After enrichment, the nucleus proteins were mainly enriched in biological process terms of cellular nitrogen compound metabolism, heterocycle metabolism, nucleobase-containing compound metabolism, cellular aromatic compound metabolism and organic cyclic compound metabolism, molecular function terms of nucleotide binding, and cellular component terms of nucleus and nucleolus (Figure 7A); the MM proteins were significantly enriched in molecular function terms of nucleotide binding (Figure 7B); the cytosol proteins were markedly enriched in biological process terms of small molecule metabolism (Figure 7C); and the ER proteins were mainly involved in vesicle and plasma membrane (Figure 7D). Furthermore, homologs of the silkworm MM proteins searched in the fly MitoMax (Figure 4C) were retrieved to validate GO enrichment, which results showed that the fly homologs were enriched in mitochondrial component (Figure 7E).

### 2.7. SilkOrganPDB Architecture and Implementation

**Figure 8:**
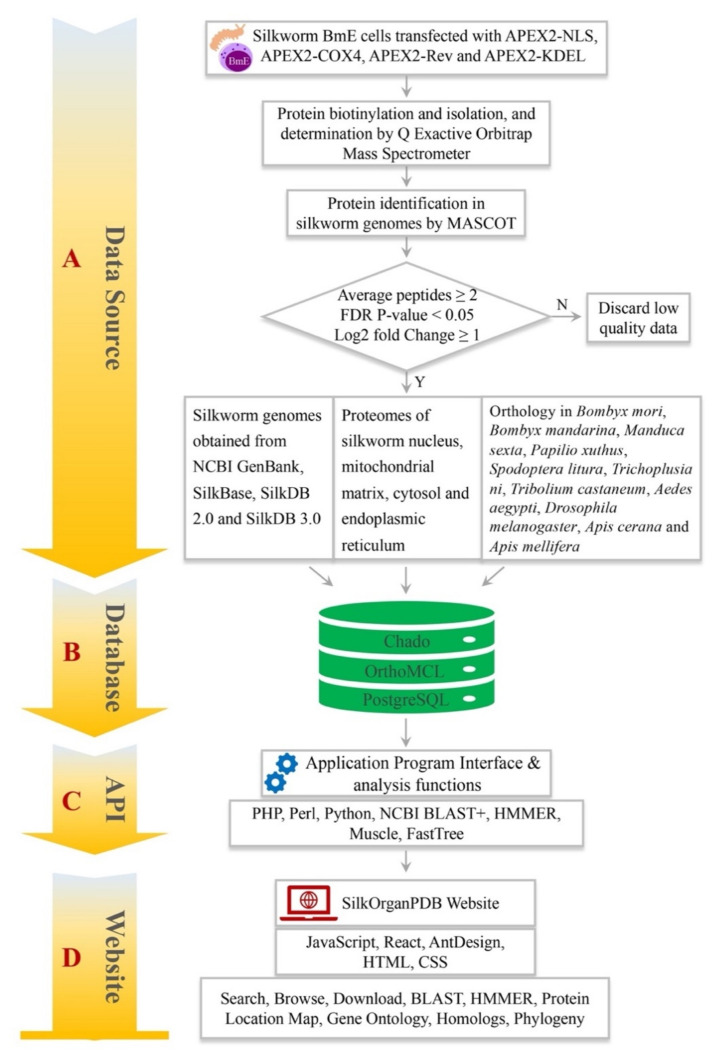
Workflow to build the SilkOrganPDB. (**A**) Isolation and identification of organelle proteins from silkworm nucleus, MM, cytosol, and ER. (**B**) Database schema of SilkOrganPDB. (**C**) Development of application program interface (API) and analysis tools. (**D**) Design of SilkOrganPDB website.

The identified organelle proteins and silkworm genome datasets obtained from GenBank, SilkBase, SilkDB 2.0 and SilkDB 3.0 were parsed and imported into the Chado schema, which is managed by the PostgreSQL (Figure 8A). Homologous proteins among *B. mori*, *B. mandarina*, *M. sexta*, *P. xuthus*, *S. litura*, *T. ni*, *T. castaneum*, *A. aegypti*, *D. melanogaster*, *A. cerana* and *A. mellifera* were predicted with the OrthoMCL, and also imported into the database (Figure 8B). Application programming interface (API) and analysis tools were developed using PHP, Perl, and Python to search data in Chado, and execute analysis programs such as NCBI BLAST+, HMMER, and FastTree (Figure 8C). Based on the Chado and API, the SilkOrganPDB website was designed with JavaScript, React, AntDesign, HTML, and CSS. The website provides users an interface to perform browse, text search, homologous sequence search, download, and phylogenetic analysis (Figure 8D).

**Figure 9:**
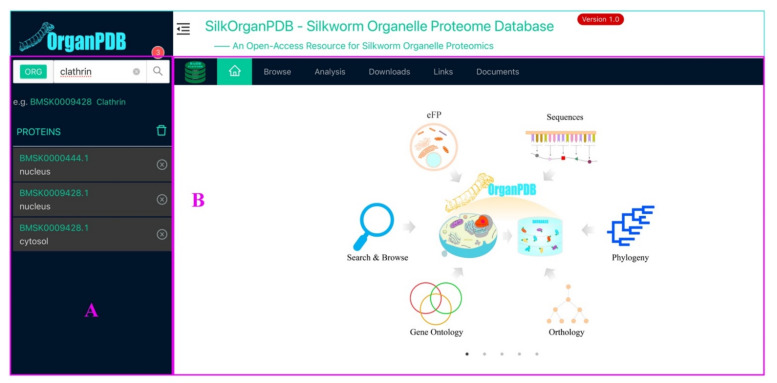
The homepage of the SilkOrganPDB. (**A**) Query panel. (**B**) Functional module panel.

The SilkOrganPDB website (https://silkorgan.biodb.org/, accessed on 30 March 2021) comprises two main panels: the query and functional modules (Figure 9). The query panel provides the user an interface to search organelle proteins by entering keywords and selecting the organelles of an organism, the results of which would be presented right below the input box (Figure 9A). The functional module panel allows the user to browse organism data, download datasets, and analyze data (Figure 9B).

#### 2.7.1. Search and Browse Modules

**Figure 10:**
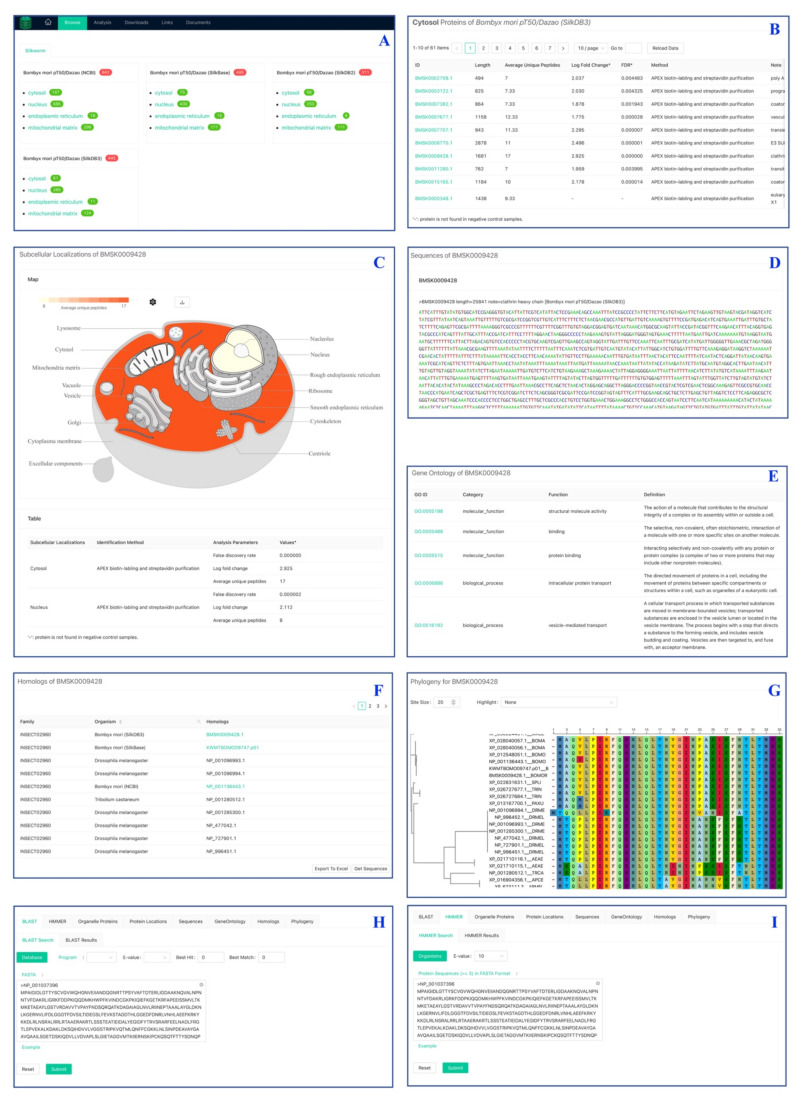
The main functional modules in the SilkOrganPDB. (**A**) Index organelle proteomes. (**B**) Browse organelle proteome. (**C**) eFP. (**D**) Sequence features. (**E**) Gene ontology. (**F**) Protein homologs in insects. (**G**) Protein phylogeny. (**H**) BLAST. (**I**) HMMER.

The organelle proteins in the database can be searched by typing protein identifiers or text keywords in the input box (Figure 9A). The query results would be shown in a table, items in which could be exported to an Excel table and checked to be listed below the input box. On the other hand, the proteome of an organelle can be browsed by clicking the name listed on the index page (Figure 10A). All identified proteins of the selected organelle would be listed in a table, which shows basic information about the protein annotation and identification (Figure 10B). Clicking on a protein ID listed in the querying results and proteome table would initiate analysis of the selected protein.

#### 2.7.2. eFP Module

The subcellular localization of a protein is shown as an electronic fluorescent pictograph (eFP) (Figure 10C), which responsively colors the organelles based on the average number of unique peptides determined by the mass spectrometry. The eFP map is drawn as a vector image, which can be magnified indefinitely and downloaded for further editing to make high-resolution figures. The color of the eFP map is configurable in absolute and relative color mode. The relative mode can be used to compare the relative quantities among different proteins.

#### 2.7.3. Sequence and GO Module

The Sequence module shows basic information for a protein, including sequences of gene, mRNA and protein, and sequence length and annotation (Figure 10D). The GO module provides the ontology terms for the protein queried, including molecular function, biological process, and cellular component (Figure 10E). By clicking on a GO ID, users can visit the detailed term information in the AmiGO database [33].

#### 2.7.4. Homologs and Phylogeny Module

The homologs module provides homology information of a *B. mori* protein in *B. mandarina*, *M. sexta*, *P. xuthus*, *S. litura*, *T. ni*, *T. castaneum*, *A. aegypti*, *D. melanogaster*, *A. cerana*, and *A. mellifera* (Figure 10F). The information table can be exported to an Excel file. All homologous sequences can be exported by clicking on the Get Sequences button. The phylogeny module performs phylogenetic analysis and constructs a phylogenetic tree based on the homologous sequences in the homologs module (Figure 10G).

#### 2.7.5. BLAST Search and HMMER Search Modules

The BLAST Search built with the NCBI BLAST+ is used to search organelle proteins in the databases for query sequences that users enter in FASTA format (Figure 10H). The search results would be shown in a table, displaying detailed alignment information for all target sequences. The HMMER Search developed with HMMER software is designed for searching homologous proteins with user-provided seed sequences (Figure 10I). The target proteins identified in the organelles would be shown as a hyperlink, by which users can explore protein features and perform analysis of them.

## 3. Discussion

The silkworm *B. mori* is an economic insect and a great model for studying lepidoptera with plenty of mutant phenotypes and fine genomic and genetic maps, as well as massive functional omics data including transcriptomes, tissue proteomes, and epigenomes. However, little proteomics data were available at the organelle level in silkworms. This was mainly limited by the lacking effective technology for targeting isolation of proteins from subcellular organelles. For example, Wang et al. identified only 87 proteins from the cytosol, mitochondria, and microsomes of silkworm midguts using two-dimensional gel electrophoresis [23]. Instead, with the APEX technology, we obtained many more organelle proteins from only one type of cell line. Therefore, it is necessary to perform large-scale isolation and identification of organelle proteomes from silkworm cells, tissues, and organs using the APEX, to make the silkworm a better lepidopteran model.

Some proteins we identified are present in multiple organelles (Figure 4B). This was also found in *Caenorhabditis elegans* organelle proteins identified by Reinke et al. [31]. Out of 3180 proteins identified in *C. elegans* cytosol and nucleus, 2048 showed multiple subcellular location (MSL), leaving only 1132 that are organelle-specific. On the one hand, the MSL indicates protein translocation among organelles. For example, the tumor suppressor factor p53 keeps a balance between the cytosol and nucleus under normal circumstances, but aggregates in the nucleus during cell apoptosis [34]. On the other hand, the MSL suggests potential protein interactions. This is especially common for hub proteins, which interact with numerous partners [35]. In ComPPI v2.1.1, the compartmentalized protein-protein interaction database, 1413 out of 1989 mitochondrial proteins of *D. melanogaster* display MSL [36]. We found 58 silkworm MM proteins homologous to the fly proteins with MSL (Table S7). The silkworm proteins with MSL provide clues for understanding their function and interplay. Although the transfection validation showed high accuracy (Figure 5), a low false-positive rate might exist, as shown in the study by Chen et al. [29]. This is probably caused by the defects of streptavidin purification. High throughput of transgenic verification is necessary to ultimately confirm the protein localizations, even though not all proteins could express in the transfected cells.

The SilkOrganPDB is the first database that provides silkworm organelle proteomes identified with an organelle-targeted method. The SilkDB 3.0 is a comprehensive data source for silkworm genomics [37]. However, the protein subcellular localizations in this database were predicted using bioinformatic methods and lack experimental verification. In the future, we will keep identifying silkworm organelle proteomes using the APEX, and developing the SilkOrganPDB for integrating more data determined experimentally and predicted with bioinformatics methods. The SilkOrganPDB provides a proteomics resource that is helpful for understanding gene functions and the better modeling of the silkworm.

## 4. Materials and Methods

### 4.1. Construction of APEX-Transgenic BmE Cell

Silkworm *B. mori* embryo cells (BmE) were cultured in Grace media containing 10% fetal bovine serum (Gibco, Australia Origin), 50 μg/mL streptomycin, and 50 unites/mL penicillin at 28 °C. The coding sequence of APEX fused with an organelle-targeting sequence, and a FLAG-tag was synthesized and cloned to a piggyBac vector for transfecting BmE cells. The amino acid sequences of nuclear localization signal (NLS) and nuclear export signal (Rev) were PKKKRKV and LQLPPLERLRLD. The MM targeting sequence was the first 30 amino acids of silkworm COX4 protein (GenBank accession no. NM_001079652.1), and the ER retention sequence was KDEL. BmE cells expressing the APEX fusion proteins were screened with the geneticin G418.

### 4.2. Biotinylation of Organelle Proteins

The transgenic cells were incubated with culture media containing biotin-phenol (final concentration 500 µM) at 28 °C for 30min. Protein-labeling was initiated by adding 1 mM H_2_O_2_ for 1 min with shaking gently. After a 1 min reaction, the quencher solution (the final concentration of 5 mM Trolox and 10 mM sodium ascorbate in DPBS) was added to halt the labeling. The cells were washed three times with 5 mM Trolox and 10 mM sodium ascorbate in DPBS.

### 4.3. Western Blotting and Streptavidin Blotting

The transgenic cells were collected and lysed with RIPA lysis buffer for 10 min at 4 °C to detect the expression of APEX fusions using Western blotting. The APEX-mediated biotinylation was verified by streptavidin blotting. The cells were collected by centrifugation at 3000× *g* for 5 min and stored at −80 °C. The cell pellet was lysed with RIPA lysis buffer (Sigma, Shanghai, China), Lot # SLBQ9770V) by additionally adding buffers including 10 mM sodium azide, 10 mM sodium ascorbate, 5 mM Trolox, 1× protease cocktail, and 1 mM PMSF at 4 °C for 10 min, and centrifuged at 14,000× *g* for 15 min at 4 °C. The protein in the supernatants was checked with 10% SDS-PAGE gel, transferred to a PVDF membrane at constant voltage 25 v for 23 min and blocked with 5% skimmed milk in PBS-buffer saline at 37 °C for 1 h. The blots were immersed in a 1:1000 dilution of anti-FLAG at 4 °C overnight and incubated in 1:5000 peroxidase labeled goat anti-mouse IgG for 1 h at 37 °C or just immersed in a 1:1000 streptavidin-HRP at 37 °C for 1 h after blocking. Before imaging using an Azure Biosystems C300, the blots were incubated with ECL Western Blotting Substrate (Bio-Rad) for 1 min.

### 4.4. Immunofluorescence Assay (IFA) of APEX Location and Biotinylated Proteins

The cells were plated into a 12-well plate for one day, followed by staining with MitoTracker Deep Red and ER dyes at 28 °C for 20 to 30 min, and then fixed in 4% paraformaldehyde at room temperature for 15 min. After fixation, the cells were washed with PBS three times for 5 min each time, permeabilized with 0.5% TritonX-100 for 20 min, and washed three times with PBS. Then, cells were blocked with 10% goat serum and 5% BSA in PBS for 1–2 h and incubated with anti-FLAG (1:800 dilution) in PBS or blocking buffer for 2 h at room temperature. After being washed five times with PBST (0.05% Tween in PBS), the cells were incubated with Alexa Flour 488 conjugate Goat anti-Mouse IgG (1:1000 dilution) and Streptavidin-AF (Alexa Fluor)−568 (1:1000 dilution) at room temperature for 1 h, and then washed again for five times with PBST. Finally, the samples were stained with DAPI (4’6-diamidino-2-phenylindole, Sigma) at room temperature for 10–20 min. Before imaging with an Olympus FV1200 laser scanning confocal microscope, the Fluoromount™ Aqueous Mounting Medium (Sigma) was added.

### 4.5. Isolation of Biotinylated Proteins

The biotinylated proteins were isolated using streptavidin-coated magnetic beads. Before enrichment, the streptavidin-coated magnetic beads were washed twice with 1 mL RIPA lysis buffer, and 4 mg total proteins were mixed and incubated with 500 μL streptavidin-coated magnetic beads for 1 h at room temperature. Then, the beads were washed with 1 mL RIPA lysis buffer two times, and 1 M KCl, 0.1 M Na_2_CO_3_, 2 M urea for one time, respectively, and again twice with RIPA lysis buffer to elute the non-specific binding proteins. Biotinylated proteins were eluted by adding 60 μL SDT buffer (4% SDS, 100 mM Tris-HCl, 1 mM DDT pH 7.6) and boiling for 5–10 min. 5 μL supernatant was used for Western blotting and the rest for mass spectrometry.

### 4.6. Identification of Biotinylated Proteins

Protein digestion was performed according to the FASP procedure described in [38]. Briefly, the protein pellet (about 30 μg) was solubilized in 30 μL SDT buffer (4% SDS, 100 mM DTT, 150 mM Tris-HCl (pH 8.0)) at 90 °C for 5 min. The detergent, DTT, and other low-molecular-weight components were removed using 200 μL UA buffer (8 M Urea, 150 mM Tris-HCl pH 8.0) by repeated ultrafiltration (Microcon units, 30 kD). Then, 100 μL 0.05 M iodoacetamide in UA buffer was added to block reduced cysteine residues and the samples were incubated for 20 min in darkness. The filter was washed with 100 μL UA buffer three times and then 100 μL 25 mM NH_4_HCO_3_ twice. Finally, the protein suspension was digested with 2 μg trypsin (Promega) in 40 μL 25 mM NH_4_HCO_3_ overnight at 37 °C, and the resulting peptides were collected as a filtrate.

LC-MS/MS determination was performed using a Q Exactive mass spectrometer (Thermo Scientific). The mass spectrometer was operated in positive ion mode. MS data was acquired using a data-dependent top10 method dynamically choosing the most abundant precursor ions from the survey scan (300–1800 m/z) for HCD fragmentation. The MS spectra data were then searched using MASCOT (Matrix Science, London, UK; version 2.2) against silkworm proteins, which were downloaded from databases of the GenBank (https://www.ncbi.nlm.nih.gov/genome/?term=ASM15162v1 (accessed on 15 October 2020)), SilkBase [4], SilkDB 2.0 [39] and SilkDB 3.0 [37], respectively. Proteins were identified with the following parameters: peptide mass tolerance = 20 ppm, MS/MS tolerance = 0.1 Da, Enzyme = Trypsin, Missed cleavage = 2, Fixed modification = Carbamidomethyl (C), Variable modification = Oxidation (M).

The protein candidates identified were then statistically screened using qprot_v1.3.5 [40]. The peptide spectral count of each protein was used to calculate the false discovery rate (FDR) *P*-value and log2 fold change (LFC) between the non-APEX and APEX-transgenic samples. Then, proteins were filtered with the following criteria: at least 2 unique peptides detected in the APEX-transgenic samples, and no peptide determined in the control samples otherwise FDR *p*-value < 0.05 and LFC ≥ 1, which means if a peptide is detected in the non-APEX samples the protein should have an FDR *P*-value less than 0.05 and LFC equal or greater than 1.

To verify subcellular locations, we randomly selected candidates from the identified proteins. Transfection vectors of pSL1180 with a fusion of the candidate protein and EGFP were constructed. Silkworm BmE cells were seeded into a 12-well plate by 5×10^5^ cells per well. 1 µg constructed vectors were transiently transfected into the BmE cells according to the instructions of X-tremeGENE HP DNA transfection reagents (Roche, product number: XTGHP-RO). The medium was replaced with fresh Grace’s Insect medium contains 10% serum after 5hours. Two days later, the transfected cells were stained with DAPI to label the nucleus and then examined by confocal microscopy (Olympus, FV1200).

### 4.7. Homologous Search in Mitochondrial Protein Databases and Insect Proteomes

The identified mitochondrial proteins in silkworm proteomes from GenBank, SilkBase, SilkDB 2.0, and SilkDB 3.0 were searched for homologs in fly, human, and mouse using BLASTP [41] with an E-value cutoff less than 1e−5 against mitochondrial protein databases of fly MitoMax [29], Human MitoCarta3.0 and Mouse MitoCarta3.0 [42], respectively. The MitoMax is a database for fly mitochondrial proteins, containing 5128 candidates identified by APEX labeling and isolation methods. The MitoCarta3.0 is an inventory of 1136 human and 1140 mouse proteins with the strong support of mitochondrial localization. From the BLASTP results, homologs of silkworm proteins in fly, human, and mouse were identified and counted.

Sequences of *D. melanogaster* proteins with MSL in ComPPI v2.1.1 [36] were retrieved from the UniProt [43]. The identified mitochondrial proteins of silkworm were aligned against the fly proteins with MSL using the BLASTP with E-value < 1e−5.

To find orthologs of silkworm proteins in other insects, we downloaded the genome data of *Bombyx mandarina* [44], *Manduca sexta* [45], *Papilio xuthus* [46], *Spodoptera litura* [47], *Trichoplusia ni* [48], *Tribolium castaneum* [49], *Aedes aegypti* [50], *D. melanogaster* [51], *Apis cerana* [52], and *Apis mellifera* [53] from GenBank. All protein sequences were all-to-all aligned using BLASTP [41] with an E-value cutoff less than 1e−5. The BLASTP results were parsed and imported into a MySQL database, tables in which were created by the OrthoMCL [54] for identifying homologs with thresholds of the percentMatchCutoff = 50 and evalueExponentCutoff = 1e−6.

### 4.8. Gene Ontology Enrichment

The identified organelle proteins were mapped to the Entrez Gene database in NCBI to retrieve ENTREZ IDs, with which gene ontology (GO) for each ENTREZ ID was retrieved from a local annotation database of *Bombyx mori* constructed with the AnnotationHub in Bioconductor [55], which is an R framework for bioinformatics and computational biology. The GOs were then enriched using the enrichGO in clusterProfiler software [56] with thresholds of pvalueCutoff = 0.05 and qvalueCutoff = 0.2. The GO terms of fly proteins were enriched by assigning the parameter “OrgDb = org.Dm.eg.db” when running the enrichGO. Subsequently, the enrichment was plotted using the dotplot in the clusterProfiler.

### 4.9. Development of Silkworm Organelle Proteome Database (SilkOrganPDB)

The genomic datasets of *Bombyx mori* pT50/Dazao downloaded from the GenBank, SilkBase, SilkDB 2.0, and SilkDB 3.0 were parsed into the Generic Feature Format version 3 (GFF3, https://github.com/The-Sequence-Ontology/Specifications/blob/master/gff3.md (accessed on 15 October 2020)) according to the Sequence Ontology [57] using the BioPerl script bp_genbank2gff3.pl (https://metacpan.org/pod/distribution/BioPerl/bin/bp_genbank2gff3 (accessed on 15 October 2020)). Then, the data in GFF3 was imported into the Chado [58], which is a relational database schema managed by the PostgreSQL and widely used to store biological data from a wide variety of organisms. The organelle proteins identified in the four versions of silkworm genomes were imported into the Chado schema as gene features, including information about the experiment, sampling, determination method, subcellular localization, and quantity.

The SilkOrganPDB server is implemented on a platform mainly composed of Linux CentOS Server 7.9 (https://www.centos.org (accessed on 10 October 2020)), PostgreSQL 9.6 (https://www.postgresql.org (accessed on 10 October 2020)), MySQL 5.7 (https://www.mysql.com (accessed on 10 October 2020)), Apache 2.4 (https://www.apache.org (accessed on 10 October 2020)), PHP 7.1, Perl 5.16 (https://www.perl.org (accessed on 10 October 2020)), and Python 3.6 (https://www.python.org/ (accessed on 10 October 2020)). The platform is installed in a computer cluster composed of Dell PowerEdge R920, which provides 1 TB random access memory and 22 TB hard drive capacity. The high performance of the system promises a high speed of analysis on big datasets.

The SilkOrganPDB website was developed using React (https://reactjs.org (accessed on 20 October 2020)), which is a JavaScript library supplying a variety of packages and plugins for rendering data and producing friendly interfaces. The BLAST tool was built with NCBI BLAST+ 2.11.0 [41], which supports searching against selectable and multiple databases. The other homologous search tool was built using the HMMER [59], which implements methods using probabilistic models known as profile hidden Markov models. The phylogenetic tool was developed by aligning homologous protein sequences with muscle [60] and then building phylogeny using FastTree [61] with default parameters, the results of which were visualized using software GraPhlAn [62].

## Data Availability

The mass spectrometry proteomics data have been deposited to the ProteomeXchange Consortium (http://proteomecentral.proteomexchange.org (accessed on 6 May 2021)) via the iProX partner repository [63] with the dataset identifier PXD025816. Other data that support the findings of this study are available from the corresponding author upon reasonable request.

## Supplementary Materials

The following are available online at https://www.mdpi.com/article/10.3390/ijms22095051/s1, Table S1: The identified nucleus proteins in silkworm BmE cells with engineered ascorbate peroxidase, Table S2. The identified mitochondrial matrix proteins in silkworm BmE cells with engineered ascorbate peroxidase, Table S3. The identified cytosol proteins in silkworm BmE cells with engineered ascorbate peroxidase, Table S4. The identified endoplasmic reticulum proteins in silkworm BmE cells with engineered ascorbate peroxidase, Table S5. The orthology for the organelle proteins of silkworm in multiple insect genomes, Table S6. The orthology for the organelle proteins of silkworm found in all insect genomes selected, Table S7. Silkworm mitochondrial matrix proteins homologous to D. melanogaster proteins with multiple subcellular location in ComPPI v2.1.1.
